# Current Status of Angiogenesis Inhibitors as Second-Line Treatment for Unresectable Colorectal Cancer

**DOI:** 10.3390/cancers15184564

**Published:** 2023-09-14

**Authors:** Satoshi Otsu, Shuichi Hironaka

**Affiliations:** 1 Department of Medical Oncology and Hematology, Oita University Faculty of Medicine, 1-1, Idaigaoka, Hasama-machi, Yufu City 879-5593, Oita, Japan; 2 Department of Medical Oncology, Kyorin University Faculty of Medicine, 6-20-2 Shinkawa, Mitaka-shi 181-8611, Tokyo, Japan

**Keywords:** colorectal cancer, second-line treatment, angiogenesis inhibitor, VEGF-A, VEGF-D

## Abstract

**Simple Summary:**

Colorectal cancer is the third most common disease and the second most common cause of death around the world. The drug for second-line treatment is determined by the type of drug used for first-line treatment and the biomarker status. As biomarkers, the *RAS* gene, *BRAF* gene, and dMMR (mismatch repair deficient)/MSI-H (microsatellite instability-high), TMB-H (tumor mutation burden-high), and HER2 statuses have been evaluated in clinical practice, and the corresponding molecularly targeted therapeutic agents should be selected based on the biomarker status. If all of these biomarkers are negative, an angiogenesis inhibitor is often used as second-line treatment. Although no useful biomarkers have been established for the selection of bevacizumab (BEV), ramucirumab (RAM), or aflibercept (AFL), which are the angiogenesis inhibitors used in second-line treatment, previous biomarker studies have suggested that VEGF-A and VEGF-D might be potential predictors of their therapeutic efficacy. The rationale for selecting these three angiogenesis inhibitors in second-line treatment should be clarified.

**Abstract:**

Colorectal cancer is the third most common disease and the second most common cause of death around the world. The drug for second-line treatment depends on the drugs used in first-line treatment and the biomarker status. As biomarkers, the *RAS* gene, *BRAF* gene, and dMMR/MSI-H, TMB-H, and HER2 statuses have been established in clinical practice, and the corresponding molecularly targeted therapeutic agents are selected based on the biomarker status. Given the frequency of biomarkers, it is assumed that when patients move on to second-line treatment, an angiogenesis inhibitor is selected in many cases. For second-line treatment, three angiogenesis inhibitors, bevacizumab (BEV), ramucirumab (RAM), and aflibercept (AFL), are available, and one of them is combined with cytotoxic agents. These three angiogenesis inhibitors are known to inhibit angiogenesis through different mechanisms of action. Although no useful biomarkers have been established for the selection of angiogenesis inhibitors, previous biomarker studies have suggested that angiogenesis-related factors such as VEGF-A and VEGF-D might be predictors of the therapeutic efficacy of angiogenesis inhibitors. These biomarkers are measured as protein levels in plasma and are considered to be promising biomarkers. We consider that the rationale for selecting among these three angiogenesis inhibitors should be clarified to benefit patients.

## 1. Introduction

Colorectal cancer was the third most common disease and the second most common cause of death around the world in 2020 [1], and approximately 20% of cases are unresectable [2]. Unresectable colorectal cancer is difficult to cure, but advances in drug therapy have led to a median survival of over 30 months [3,4]. Chemotherapy for unresectable colorectal cancer is expected to lead to long-term survival by a good sequential baton pass from first-line treatment to the later lines [5]. Progression-free survival (PFS) in the first-line treatment for unresectable colorectal cancer is approximately 10 months, and PFS in the second-line treatment is reported to be about 6 months, with overall survival (OS) of 10–13 months, both of which are still unsatisfactory [6,7,8]. Improving the outcome of second-line treatment is considered an important issue that could contribute to the survival of patients with colorectal cancer.

The history of second-line treatment for unresectable colorectal cancer dates back to a 1998 report [9]. A comparison between irinotecan and best supportive care was made in patients with unresectable colorectal cancer who had failed fluorouracil. The primary endpoint, the 1-year survival rate, was significantly better in the irinotecan group than in the best supportive care group (36.2% vs. 13.8%), showing the survival benefit of second-line treatment for unresectable colorectal cancer. Subsequently, a comparative study investigating the order of administration of FOLFOX and FOLFIRI, which had been established as the standard first-line treatment regimen, was conducted. The results showed quite similar efficacy between FOLFOX followed by FOLFIRI and FOLFIRI followed by FOLFOX [5]. This also demonstrated the usefulness of FOLFOX or FOLFIRI as second-line treatment. Next came molecularly targeted therapies such as bevacizumab (BEV), an angiogenesis inhibitor, and anti-EGFR antibodies. As second-line therapy, adding BEV to FOLFOX showed an overall survival (OS) benefit compared with FOLFOX therapy [10]. In terms of anti-EGFR antibodies, the EPIC trial failed to show the OS benefit of cetuximab (Cmab) plus IRI vs. IRI alone in patients with colorectal cancer expressing EGFR [11]. On the other hand, the 20050181 study showed significant OS and PFS benefits of panitumumab (Pmab) plus FOLFIRI vs. FOLFIRI for *KRAS*-wild colorectal cancer, whereas no OS benefit was observed for *KRAS*-mutant colorectal cancer [12] (Table 1). These results showed that adding anti-EGFR antibodies to chemotherapy was beneficial for *KRAS*-wild colorectal patients even in the second-line setting.

In the current second-line treatment, in addition to *RAS* mutation and *BRAF* mutation, the dMMR/MSI-H, HER2, and TMB-H statuses are also important in selecting treatment regimens, and the drug of choice depends on the regimen used in first-line treatment. In colorectal cancer, the incidence of *RAS* wild-type is reported to be 40–50% [13,14,15], *BRAF V600E* mutation about 10% [16], dMMR/MSI about 15% [17,18], TMB-H about 11% [19], and HER2-positive 2–11% [20]. Depending on the biomarker status, a decision regarding whether to select an anti-EGFR antibody, BRAF inhibitor, MEK inhibitor, HER2 inhibitor, or immune checkpoint inhibitor should be made. In patients with *RAS* mutation, an anti-EGFR antibody is not recommended by regional guidelines as one of the standard treatments [21,22,23]. In patients with the *BRAF V600E* mutation, encorafenib plus binimetinib plus Cmab or encorafenib plus Cmab is recommended [24]. In cases of HER2-positive (IHC3+ or IHC2+/FISH-positive) colorectal cancer, trastuzumab plus pertuzumab therapy is recommended [25]. In cases with TMB-H cancer, pembrolizumab is recommended [26], and in cases with dMMR/MSI-H, ipilimumab plus nivolumab therapy is recommended [27].

Currently, three anti-angiogenesis inhibitors are available for metastatic colorectal cancer in the second-line setting. BEV, ramucirumab (RAM), or aflibercept (AFL) can be used regardless of prior BEV use in second-line treatment (Table 2). Based on the frequency of biomarkers, it is estimated that angiogenesis inhibitors are the most commonly used in second-line treatment. These three angiogenesis inhibitors are known to inhibit angiogenesis through different mechanisms of action. Previous biomarker studies suggested that angiogenesis-related factors such as VEGF-A and VEGF-D might be potential predictors of the therapeutic efficacy of angiogenesis inhibitors [28,29,30,31,32,33,34]. Currently, a prospective study to compare the safety and efficacy across these three angiogenesis inhibitors combined with FOLFIRI is ongoing in Japan, and it is exploring biomarkers that could help to select an appropriate angiogenesis inhibitor to improve the outcomes of second-line treatment [35].

In this review, the second-line treatment for metastatic colorectal cancer is outlined with a focus on these angiogenesis inhibitors.

## 2. Second-Line Treatment with Angiogenesis Inhibitors for Unresectable Colorectal Cancer

### 2.1. Angiogenic Factors and Angiogenesis Inhibitors

Vascular endothelial growth factor (VEGF) is known to play an important physiological role in embryogenesis, wound healing, and angiogenesis, and it is associated with malignant tumor growth, invasion, and metastasis pathologically [36,37]. Although the term VEGF usually refers to VEGF-A, there are other isoforms, such as VEGF-B, VEGF-C, VEGF-D, and placental growth factor (PlGF), which are collectively known as the VEGF family. These family members have been found to differ in expression patterns, receptor affinity, and biological functions [38].

VEGF-A is the most important angiogenic factor [39]. Variants of VEGF121, VEGF145, VEGF148, VEGF165, VEGF183, VEGF189, and VEGF206 are known to exist due to selective splicing and have different affinities for the receptor [37]. The most frequently expressed variants are VEGF121, 165, and 189, of which VEGF165 is the most frequently expressed and has the strongest angiogenic effect [40]. VEGF-A binds to vascular endothelial growth factor receptor 1 (VEGFR-1) and VEGFR-2. VEGFR-1 is considered to have high affinity for VEGF-A. However, it has low tyrosine kinase activity and is considered a decoy receptor [41].

VEGF-D is a protein similar to VEGF-C; it plays a central role in lymphangiogenesis, and it is also considered essential for angiogenesis. It is expressed at high levels in the lungs, the site of lymphatic vessel development in the fetus, as well as in adults, and it is highly expressed in the heart, lungs, skeletal muscle, and small intestine [42]. It binds with strong affinity to VEGFR-2 and VEGFR-3. Although much of its function has not been elucidated, it has been reported to be associated with lymphangiogenesis and metastasis within tumors [43].

BEV is a humanized anti-VEGF IgG1 monoclonal antibody based on murine anti-VEGF monoclonal antibody muMAb A4.6.1, which selectively binds to VEGF-A [44]. VEGF-A is a major regulator of angiogenesis and is upregulated in most human tumors, contributing to tumor growth and metastasis. BEV selectively binds to VEGF-A, thereby blocking the binding of VEGF-A to its receptors (VEGFR-1 and VEGFR-2) expressed on vascular endothelial cells, blocking the VEGF signaling pathway and inhibiting VEGF-induced angiogenesis in tumor tissue [45,46,47]. It is the first anti-cancer drug in the world to demonstrate clinical usefulness as an angiogenesis inhibitor [48,49].

RAM is a human anti-VEGFR-2 monoclonal antibody (IgG1b) against vascular endothelial growth factor receptor 2 (VEGFR-2a) that binds to VEGF-A, VEGF-C, and VEGF-D. In preclinical studies, RAM has been shown to bind specifically to human VEGFR-2 with high affinity, inhibiting the binding of VEGF ligands (VEGF-A, VEGF-C, and VEGF-D) to VEGFR-2 and blocking VEGFR-2 activation, thus suppressing tumor tissue angiogenesis [50,51,52].

AFL is a dimeric glycoprotein with a molecular weight of 97 kDa, produced by fusing the second immunoglobulin (Ig)-like C2 domain of human VEGFR-1 with the third Ig-like C2 domain of human VEGFR-2, which is then fused to the stationary region (Fc domain) of human IgG1 [53]. It binds to VEFG-A, VEGF-B, and PlGF, leading to the suppression of tumor growth by inhibiting vascularization.

### 2.2. Availability of Angiogenesis Inhibitors for Second-Line Treatment

The triple combination therapy of BEV combined with oxaliplatin and fluoropyrimidine was one of the standard first-line treatments for unresectable colorectal cancer and is the most frequently used regimen worldwide [54]. Following the approval of BEV, a large, prospective, observational study in the USA identified the continuous use of BEV as an independent factor prolonging survival in second-line colorectal cancer patients who experienced progression on first-line BEV treatment [55]. Subsequently, a clinical trial was conducted to evaluate the benefit of the continuous administration of BEV after the failure of first-line BEV combination treatment. A phase III trial (ML18147) demonstrated the add-on effect of BEV with FOLFOX or FOLFIRI in colorectal cancer patients who failed the triplet therapy containing BEV, establishing it as the standard of care for second-line colorectal cancer therapy [6]. Subsequently, the RAISE and VELOUR trials showed the add-on effects of RAM and AFL with FOLFIRI in the second-line setting, respectively [7,8]. On the basis of these results, RAM plus FOLFIRI and AFL plus FOLFIRI were established as standard second-line treatments for colorectal cancer, as well as BEV plus FOLFIRI [21,22,23]. The results of these three trials are shown in Table 3.

Notably, patients who failed in the early period of first-line treatment were excluded from the ML18147 study, and patients who had not received BEV in first-line treatment, accounting for 30% of all participants, were included in the VELOUR study. Although the eligibility criteria in each study were somewhat different, they all demonstrated the usefulness of an angiogenesis inhibitor as second-line treatment. However, no head-to-head randomized studies have been conducted to compare the efficacy and safety of these three angiogenesis inhibitors, and there is no evidence to support their choice in routine practice.

The 2019 edition of the Japanese guidelines for the treatment of colorectal cancer for physicians [23] strongly recommends the combination of FOLFIRI therapy and one of the angiogenesis inhibitors, BEV, RAM, or AFL, as standard second-line treatment after oxaliplatin combination therapy. The recommendations are comparable among the three angiogenesis inhibitors, and their selection should be based on the risk–benefit balance, such as the toxicity profile and medical costs. The NCCN Guidelines version 1, 2022 [21] recommend the combination of FOLFIRI therapy and an angiogenesis inhibitor as second-line treatment after concurrent oxaliplatin, and, when any of the three angiogenesis inhibitors is used, BEV is preferred in terms of toxicity and cost. Referring to the literature cited for cost [56], RAM was the most expensive in the USA, albeit as of 2015. Similarly, the ESMO guidelines [22] recommend the combination of FOLFIRI therapy and an angiogenesis inhibitor as standard second-line treatment after oxaliplatin combination therapy. For patients who have not received BEV as first-line treatment, the combination of BEV or AFL (limited to FOLFIRI) is recommended, and for patients who have received BEV as first-line treatment, all three drugs (BEV, RAM, and AFL) are recommended, with RAM and AFL being especially recommended in early refractory cases. These recommendations are thought to be based on the conditions of the subject populations in the three studies mentioned above, which demonstrated the benefit of each, but the medical biological reasons for these recommendations are not clear.

The toxicities from the three pivotal phase III studies are summarized in Table 4. The frequency of occurrence of these adverse events gives the impression that the toxicity of BEV is mild, but since the different trials are arranged side by side, the results should be interpreted with caution. Regarding the cost in Japan, the combination of RAM plus FOLFIRI is the most expensive when estimated based on the average body surface area of Japanese patients (Table 4).

Thus, three angiogenesis inhibitors have demonstrated utility and are available for use as second-line treatments for colorectal cancer, but a strategy for selection among them in terms of therapeutic efficacy remains unestablished.

### 2.3. Biomarkers for Angiogenesis Inhibitors

Recently, angiogenesis-related factors have been reported as possible biomarkers of efficacy for BEV, RAM, and AFL.

#### 2.3.1. Biomarkers for BEV

A meta-analysis reported in 2013 analyzed clinical trials (one report for colorectal cancer, two for lung cancer, one for renal cell carcinoma) in which VEGF-A was measured by ELISA in pretreatment plasma samples. The median baseline measurements were 44 pg/mL, 36 pg/mL, 45 pg/mL, and 55 pg/mL, respectively, in each study. In terms of distribution, almost half of the patients were in the 12.5–49 pg/mL range, with a measurable range of 12.5–889 pg/mL [57]. This analysis showed that VEGF-A is a prognostic factor, but not a predictor of BEV efficacy. A 2013 report from the MD Anderson Cancer Center used a multiplex bead assay to measure VEGF-A levels in plasma samples from the untreated group, the progression group after chemotherapy without BEV, and the progression group after chemotherapy with BEV, with median values of 760 pg/mL, 534 pg/mL, and 1740 pg/mL, respectively [58].

Bai et al. reported a retrospective study analyzing angiogenesis-related factors in the pretreatment plasma of patients with unresectable colorectal cancer undergoing first-line treatment [28]. The median PFS of the chemotherapy group with BEV vs. chemotherapy alone in the low VEGF-A group was 10.8 months vs. 6.4 months (hazard ratio [HR] 0.53, 95% confidence interval [CI] 0.36–0.76, *p* = 0.01), and the median OS was 50.0 months vs. 28.0 months (HR 0.42, 95%CI 0.20–0.84, *p* = 0.011). In the high VEGF-A group, median PFS was 7.8 months vs. 7.8 months (HR 0.90, 95%CI 0.53–1.53, *p* = 0.70), and median OS was 19.6 months vs. 29.9 months (HR 1.25, 95%CI 0.77–2.02, *p* = 0.37) in the chemotherapy with BEV group and chemotherapy alone group, respectively (Table 5). Although this was a retrospective study, it suggested that lower levels of VEGF-A in the pretreatment plasma may be a possible biomarker for treatment with BEV.

In addition, in a phase II study comparing BEV plus FOLFOX with BEV plus FOLFIRI in first-line treatment, both groups were divided into high and low pretreatment plasma VEGF-A groups. The OS was significantly worse in the high group than in the low VEGF-A group [29]. Another phase II study examined whether VEGF-A short isoform (VEGF-Asi) could be a predictor of BEV efficacy in BEV plus FOLFOX/XELOX in first-line treatment [30]. Pretreatment plasma samples were collected, and the pVEGF-Asi level was divided according to the median pVEGF-Asi value of 37 (range 6.5–262) pg/mL. The HR of the high pVEGF-Asi group vs. the low group for PFS was 1.3 (95%CI 0.8–2.1). These results of biomarker analyses from phase II trials are consistent with previous results showing that lower levels of VEGF-A are associated with benefits from BEV.

The MAX trial, a phase III study, demonstrated the benefit of BEV in the first-line treatment of unresectable colorectal cancer [59]. In a post hoc analysis of this trial, angiogenic factor expression in tumor tissue (immunohistochemistry) and the therapeutic effect were examined [31]. In the group with weak expression of VEGF-D (score 0, 1+), the median OS in the BEV with chemotherapy group vs. the chemotherapy alone group was ‘not reached’ vs. 18.9 months (HR 0.35, 95%CI 0.13–0.90). In the moderate expression (score 2+) group, median OS was 21.6 vs. 20.6 months (HR 0.82, 95%CI 0.52–1.30). In the strong expression (score 3+) group, median OS was 19.4 vs. 24.5 months (HR 1.28, 95%CI 0.79–2.09), respectively, indicating that the therapeutic effect of BEV was decreased as the expression of VEGF-D increased (interaction *p* = 0.01) (Table 5).

These results suggest that the therapeutic effect of BEV may be poor in the high VEGF-A and high VEGF-D expression groups before treatment, and good in the low VEGF-A and VEGF-D expression groups.

#### 2.3.2. Biomarkers for RAM

Biomarker analyses in the RAISE study examined plasma VEGF-C, VEGF-D, sVEGFR-1, sVEGFR-2, sVEGFR-3, and VEGFR-2. In the analysis of the RAISE study [32], a cutoff value of 115 pg/mL (median 135 pg/mL) was set by multivariate analysis in a predefined exploratory set of populations, and efficacy was confirmed in a validation set. Finally, analysis of the full translational research set (exploratory set plus validation set) was reported. The OS of the chemotherapy with RAM group vs. chemotherapy alone group in the high VEGF-D level was significantly better in the combined RAM group. In contrast, in the lower VEGF-D group, OS was worse in the combined RAM group. Analysis of the OS also showed an interaction between high and low VEGF-D levels. The same results were observed for PFS (Table 5). There were no significant interactions of other factors.

These results suggest that a high plasma VEGF-D level is a potential predictor of RAM efficacy; it is interesting that the effect is opposite to the trend for BEV.

#### 2.3.3. Biomarkers for AFL

In the biomarker analysis of the VELOUR study, VEGF-A, PlGF, endoglin, T-cadherin, VEGFR-3, serum amyloid P-component, vitamin D binding protein, neuropilin-1, C-reactive protein, IL-8, MIF, EoTaxin-1, VEGFR2, Hepsin, and SPD were examined [33,34]. The results showed that OS in the high plasma VEGF-A group was better in the AFL group than in the placebo group. On the other hand, there was no clear difference in the low VEGF-A group, suggesting that a high plasma VEGF-A level may be a predictor of the treatment response to AFL, with the opposite effect to the trend for BEV in VEGF-A. The overall median baseline VEGF-A level in the VELOUR trial was 144 pg/mL, which was used as a cutoff to compare efficacy (Table 5).

#### 2.3.4. Other Biomarkers Thought to Be Associated with Angiogenesis Inhibitors

PlGF, a member of the VEGF family, is a 25-kDa cytokine discovered in the human placenta that binds to VEGFR-1 and is known to be involved in angiogenesis [60]. Although PlGF as a biomarker has been studied in a phase II trial of BEV plus FOLFIRI as a first-line treatment for unresectable colorectal cancer, its potential as a predictive biomarker was not shown [61]. In the biomarker analysis of the VELOUR study described above, the OS in patients with high plasma PlGF levels was better in the AFL group than in the placebo group (12.2 months vs. 9.7 months, HR 0.586, *p* = 0.00429). On the other hand, the OS in patients with low plasma PlGF levels was comparable between the AFL and placebo groups (12.8 months vs. 11.7 months, HR 0.947, *p* = 0.653), indicating that PlGF may be a predictor of the response [34].

IL-8 is a chemokine that was first discovered as a neutrophil migration factor, but it has since been found to be associated with angiogenesis and tumor growth [62]. In the aforementioned phase II trial of BEV plus FOLFIRI, when pretreatment plasma IL-8 levels were divided into high and low, OS was better in patients with a low level vs. a high level (11.0 months vs. 15.1 months, HR 2.05, *p* = 0.037), suggesting that IL-8 might be a possible prognostic factor [61]. A post hoc analysis of the biomarker in the VELOUR trial was reported at ASCO-GI in 2015. The plasma IL-8 level was shown to be a possible prognostic factor, with OS worse in the high IL-8 group than in the low IL-8 group, regardless of the AFL combination. In the high IL-8 group alone, median OS was 9.4 months in the AFL plus FOLFIRI group and 8.0 months in the FOLFIRI group, significantly better in the AFL combination group (HR 0.632, 95%CI 0.489–0.817, *p* = 0.0006). In the low IL-8 group, there was no clear difference between patients with and without concomitant AFL (18.8 months vs. 19.8 months, HR 0.957, *p* = 0.782). These results suggest that IL-8 may be a possible predictor of AFL efficacy [33]. The RAINBOW study, which investigated the additive effects of RAM for advanced gastric cancer, showed that IL-8 might be a possible prognostic factor for RAM in patients with unresectable gastric cancer, although the cancer types were different, with lower pretreatment IL-8 levels reported to be associated with a better prognosis [63].

## 3. Angiogenesis Inhibitors and Anti-EGFR Antibodies

### 3.1. Second-Line Treatment with Anti-EGFR Antibodies

Although anti-EGFR antibodies are likely to be used in the first-line treatment of RAS wild-type, left-sided colorectal cancer, no conclusion has been reached as to whether the primary site influences the effect of anti-EGFR antibodies in previously treated patients.

A small number of retrospective studies have been reported. Reports from Taiwan of *KRAS* wild-type colorectal cancer refractory to two or three regimens [64] and from Italy of *RAS/BRAF* wild-type colorectal cancer refractory to two or three regimens [65] both suggested that the therapeutic effect of anti-EGFR antibodies is better in left-sided colorectal cancer. However, these were retrospective reports of a small number of cases and cannot be considered concrete evidence. The NCCN guidelines state that the use of anti-EGFR antibodies in previously treated cases can be considered regardless of the localization of the primary site [21].

The frequency of *RAS* mutations in colorectal cancer has been reported to be 50%–60% for *KRAS*, *NRAS*, and *HRAS* [13,14,15]. Around 80% of *RAS* wild-type patients have left-sided disease [66]. It is thought that an anti-EGFR antibody should be selected for these patients in first-line treatment. Thus, the use of anti-EGFR antibodies in second-line treatment is expected to be limited to approximately 10% of colorectal cancers.

As a second-line treatment for *RAS/BRAF* wild-type colorectal cancer, treatment regimens using anti-EGFR antibodies are recommended by regional guidelines as one of the standard treatments [21,22,23]. The approved anti-EGFR antibodies are Cmab, a chimeric antibody, and Pmab, a fully human antibody.

In 2008, Sobrero et al. reported the EPIC trial comparing Cmab plus irinotecan vs. irinotecan alone in patients with colorectal cancer who were refractory to the combination of fluoropyrimidine and oxaliplatin (with or without BEV) [11]. Although the primary endpoint of OS did not show superiority, it was considered to be influenced by the fact that 47% of patients in the irinotecan monotherapy group received Cmab as subsequent treatment, and the usefulness of Cmab as second-line treatment was taken to have been demonstrated.

For second-line treatment with Pmab, a comparative study that tested the superiority of Pmab plus FOLFIRI over FOLFIRI in metastatic colorectal cancer that was refractory to fluoropyrimidine-based first-line treatment was reported [12]. The primary endpoints were PFS and OS by *KRAS* status. *KRAS* testing was performed after the completion of patient enrollment and immediately prior to the interim OS analysis. The PFS of Pmab plus FOLFIRI was better compared with FOLFIRI in the wild-type *KRAS* population, indicating the superiority of Pmab. OS in the wild-type *KRAS* group was not shown to be statistically significantly different between the Pmab combined group and FOLFIRI alone group. The number of patients receiving BEV in first-line treatment was about 20% in both groups, and subgroup analysis showed that prior BEV treatment did not affect PFS (Table 1).

### 3.2. Angiogenesis Inhibitors vs. Anti-EGFR Antibody with Bevacizumab beyond Progression

As presented in Section 3.1, anti-EGFR antibodies have shown utility in second-line treatment. In the same way, angiogenesis inhibitors have also shown usefulness in second-line treatment, as mentioned in Section 2.2. Which of these molecularly targeted agents should we use in second-line treatment after BEV use? Several clinical trials have been conducted to address this question (Table 6).

Hecht et al. reported the results of a randomized, phase II trial comparing Pmab plus FOLFIRI with Bev plus FOLFIRI in patients with *KRAS* wild-type colorectal cancer after the failure of a BEV plus oxaliplatin-based chemotherapy regimen [67]. Although the response rate was better in the Pmab group, the primary endpoint, which was PFS, was not met. Shitara et al. compared Pmab plus FOLFIRI with BEV plus FOLFIRI as second-line treatment in patients with *KRAS* exon 2 (codon 12, codon 13) wild-type colorectal cancer who had failed first-line treatment including fluoropyrimidine, oxaliplatin, and BEV [68]. This was a randomized, phase II trial with a primary endpoint of OS. The trial was originally set up to investigate the superiority of BEV plus FOLFIRI over Pmab plus FOLFIRI, but it was changed to investigate the similar OS in both arms based on the results of several randomized trials published after the start of this trial. The HR for the primary endpoint of OS was 1.16 (95%CI 0.76–1.77), suggesting similar treatment efficacy. Bennouna et al. conducted a phase II trial comparing chemotherapy with BEV with Cmab after the failure of chemotherapy with BEV for *KRAS/NRAS* wild-type unresectable colorectal cancer [69]. The primary endpoint of 4-month PFS was 80.3% (95%CI 68.0–88.3%) in the BEV chemotherapy arm and 66.7% (95%CI 53.6–76.8%) in the Cmab arm.

These trials have not shown significant differences in primary endpoints (PFS or OS) between chemotherapy with an anti-EGFR antibody and chemotherapy with BEV after the failure of BEV combination chemotherapy. However, these were phase II trials with a small number of patients and cannot be considered definitive. More importantly, whether BEV is appropriate to compare to anti-EGFR antibodies in these situations is also inconclusive.

### 3.3. Treatment with Angiogenesis Inhibitors after Use of an Anti-EGFR Antibody

Although anti-EGFR antibodies are likely to be used in the first-line treatment of RAS wild-type, left-sided colorectal cancer, there is no clear evidence for the efficacy of angiogenesis inhibitors in subsequent second-line treatment.

An Italian, multicenter, retrospective study analyzed 277 colorectal cancer cases treated with anti-EGFR antibodies in first-line treatment and angiogenesis inhibitors in second-line treatment [70]. Of the 277 patients, 228 (82%) were treated with BEV and 49 (18%) with AFL. Although it was impossible to obtain a definitive conclusion due to the retrospective nature of the study and variability in the number of patients, a trend toward longer PFS for BEV was shown.

A retrospective report from Japan was also published [71]. An analysis of 1163 patients with wild-type RAS who were given anti-EGFR antibodies in first-line treatment was performed. Of the 1163 cases, 83% involved the left-sided colon, and 84% were given Pmab plus FOLFOX as the first-line treatment regimen. Angiogenesis inhibitors used in second-line treatment included BEV (63%), RAM (27%), and AFL (10%). This study analyzed treatment durations (from the start of second-line treatment to the end of anti-tumor drug therapies), rather than PFS. Median PFS was 12.5 months (95%CI 11.2–14.0 months) for the BEV group, 12.5 months (95%CI 11.2–14.8 months) for the RAM group, and 14.0 months (95%CI 10.4–17.0 months) for the AFL group. The results were similar in each group.

A prospective, phase II trial from Japan was reported at ASCO-GI in 2022. The efficacy and safety of RAM plus FOLFIRI in second-line treatment after the first-line use of an anti-EGFR antibody were evaluated, and PFS was reported to be 7 months (95%CI 5.7–7.6 months) [72].

Cases in which patients received anti-EGFR antibodies in the first-line treatment are expected to receive angiogenesis inhibitors in second-line treatment. Two retrospective studies and one prospective study involving such patients described above seem to suggest the usefulness of angiogenesis inhibitors [70,71,72]. Historically, the results of these studies are similar to the data of BEV beyond progression [6,7,8]. However, it is unclear which angiogenesis inhibitor should be selected in this setting.

## 4. New Angiogenesis Inhibitors and Regimens

In the area of angiogenesis inhibitors, a new drug, fluquintinib, has also been developed in the later line [73,74]. A phase II study of fluquintinib in combination with capecitabine as maintenance therapy after first-line treatment, comparing it to BEV with capecitabine, is ongoing [75]. In addition, results from a phase II study of fluquintinib in combination with an anti-PD-1 antibody in patients receiving third-line therapy have been reported, suggesting that the combination may be effective in combination with immune checkpoint inhibitors [76].

There are also a number of ongoing clinical trials using immune checkpoint inhibitors for the second-line treatment of colorectal cancer, including a phase III study of chidamide, the histone deacetylase inhibitor, and sintilimab, the anti-PD-1 antibody, in combination with BEV plus FOLFIRI, the standard of care for second-line treatment [77].

Even in the development of these new angiogenesis inhibitors and new regimens, BEV is often the drug of choice for concomitant use. This may change as the usefulness of the aforementioned biomarkers is demonstrated.

## 5. Conclusions

The second-line treatment for unresectable colorectal cancer, particularly with angiogenesis inhibitors, was reviewed. As discussed in detail in the section on angiogenesis inhibitors, there is currently no clear rationale in choosing which angiogenesis inhibitor to use in second-line treatment. This is a very troublesome situation in clinical practice. We are faced with this problem in various situations, whether it is failure to respond to BEV regimens, anti-EGFR antibody regimens, or postoperative adjuvant chemotherapy. More recently, as reported in the SUNLIGHT trial [78], the value of the concomitant use of BEV with trifluridine/tipiracil has been demonstrated in third-line treatment and beyond. In this case, too, it is unclear whether the BEV combination is better than RAM, AFL, or the same.

Each of the three angiogenesis inhibitors has a slightly different biological mechanism of action. This raises the question of whether there might be some difference in the effectiveness of treatment. To address the confusion and biological questions in clinical practice, the authors initiated a prospective clinical trial, the JCOG2004 trial, which is a randomized, phase II trial comparing BEV plus FOLFIRI with RAM plus FOLFIRI and AFL plus FOLFIRI in patients with unresectable colorectal cancer who have failed or not tolerated first-line treatment with fluoropyrimidine and oxaliplatin, and exploring biomarkers useful for treatment selection (jRCTs031220058). As mentioned in the angiogenesis inhibitor section, we expect VEGF-D and VEGF-A to be useful biomarkers and will compare the therapeutic effects of high and low VEGF-D and VEGF-A using the Angiogenesis Panel test [79]. Even if we refer to the ASCO annual meeting 2023 or Clinical Trials.gov, there are no clinical trials comparing these three angiogenesis inhibitors, and JCOG2004 is the first randomized trial in the world. We believe that, if we complete this clinical trial, we may move closer to a method of differentiating the use of angiogenesis inhibitors in the future.

As we have shown, in unresectable advanced colorectal cancer, the inhibition of angiogenesis is undoubtedly an important therapeutic strategy in all lines of treatment. It is anticipated that angiogenesis inhibitors will be used in combination with newer agents. In this important therapeutic strategy, it is vital to elucidate the predictors of therapeutic efficacy and to select the appropriate drug from among the many angiogenesis inhibitors.

## Figures and Tables

**Table 1:** Pivotal studies of second-line treatment for colorectal cancer.

Clinical Trial	Phase	Previous Regimen	RAS Status	Treatment Arm	*n*	Primary Endpoint	Median OS, Months (95%CI)	Median PFS, Months (95%CI)	ORR% (95%CI)	HR
Cunningham, D. et al. (1998) [9]	III	fluorouracil-based regimens	NE	IRI	189	OS	9.2	NE	NE	NE
				BSC	90		6.5			
Grothey, A. et al. (2004) [5] *	III	FOLFIRI	NE	FOLFOX	81			4.2 (3.7–5.2)	15(7–23)	NE
		FOLFOX		FOLFIRI	69			2.5 (2.1–3.3)	4 (0–9)	
Giantonio, B.J. et al. (2007) [10]	III	Fluoropyrimidine+ IRI	NE	BEV + FOLFOX	286	OS	12.9	7.3	22.7	OS (BEV + FOLFOX vs. FOLFOX); 0.75, *p* = 0.0011
				FOLFOX	291		10.8	4.7	8.6	
				BEV	243		10.2	2.7	3.3	
Sobrero, A.F. et al. (2008) [11]	III	Fluoropyrimidine+ oxaliplatin (with or without BEV)	EGFR expressing	Cmab + IRI	648	OS	10.7 (9.6–11.3)	4.0 (3.2–4.1)	16.4 (13.6–19.4)	OS: (0.975; 95%CI: 0.854–1.114; *p* = 0.71)
				IRI	650		10.0 (9.1–11.3)	2.6 (2.1–2.7)	4.2 (2.8–6.0)	
Peeters, M. et al. (2010) [12]	III	Fluoropyrimidine-based chemotherapy (with or without oxaliplatin, BEV)	*KRAS* wild type	Pmab + FOLFIRI	303	PFS/OS	14.5 (13.0–16.0)	5.9 (5.5–6.7)	35 (30–41)	PFS: (0.73; 95%CI: 0.59–0.90; *p* = 0.004) OS: (0.85; 95%CI: 0.70–1.04; *p* = 0.12)
				FOLFIRI	294		12.5 (11.2–14.2)	3.9 (3.7–5.3)	10 (7–14)	
			*KRAS* mutant type	Pmab + FOLFIRI	238		11.8 (19.4–13.3)	5.0 (3.8–5.6)	13 (9–18)	PFS: (0.85; 95%CI: 0.68–1.06; *p* = 0.14) OS: (0.94; 95%CI: 0.76–1.15)
				FOLFIRI	248		11.1 (10.3–12.4)	4.9 (3.6–5.6)	14 (10–19)	

BEV: bevacizumab, AFL: aflibercept, RAM: ramucirumab, OS: overall survival, PFS: progression-free survival, ORR: overall response rate, HR: hazard ratio, NE: not evaluated. * Although the study compared first-line and second-line treatments together, only the results of the second-line treatment portion are shown.

**Table 2:** Summary of recommended treatments for unresectable colorectal cancer.

First-Line		Second-Line	Third or Later
intensive therapy recommended	fluoropyrimidine+oxaliplatin +/−molecular targeted agents	**BEV + FOLFIRI**	Regorafenib or t rifluridine/tipiracil +/−BEV or Anti EGFR Ab +irinotecan
		**RAM + FOLFIRI**	
		**AFL + FOLFIRI**	
		Irinotecan +/−fluoropyrimidine +/−molecular targeted agents	
		BRAF inhitor +Anti EGFR Ab +/−MEK inhibitor	irinotecan-based therapy or late line
		HER2 inhibitor	
	fluoropyrimidine+irinotecan +/−molecular targeted agents	fluoropyrimidine+oxaliplatin +/−molecular targeted agents	Regorafenib Or trifluridine/tipiracil +/−BEV or Anti EGFR Ab +irinotecan
		BRAF inhitor +Anti EGFR A +/−MEK inhibitor	oxaliplatin-based therapy or late line
		HER2 inhibitor	
intensive therapy not recommended	fluoropyrimidine+/−BEV	to be considered depending on the patient’s condition	
	Ani EGFR Ab		
dMMR/MSI-H	pembrolizumab	intensive treatment or not intensive	

Modified quotation from Refs. [21,22,23].

**Table 3:** Pivotal studies of second-line treatment with angiogenesis inhibitors.

Clinical Trial	Treatment Arm	*n*	Primary Endpoint	Median OS, Months (95%CI)	Median PFS, Months (95%CI)	ORR, %	HR
Bennouna, J. et al. (2013) [6]	BEV+FOLFOX/FOLFIRI	409	OS	11.2 (10.4–12.2)	5.7 (5.2–6.2)	5.4	OS: (0.81; 95%CI 0.69–0.94; *p* = 0.0062)
	FOLFOX/FOLFIRI	411		9.8 (8.9–10.7)	4.1 (3.7–4.4)	3.9	
Van Cutsem, E. et al. (2012) [8]	AFL + FOLFIRI	612	OS	13.5 (12.52–14.95)	6.9 (6.51–7.2)	19.8	OS: (0.817; 95.34%CI 0.713–0.937; *p* = 0.0032)
	FOLFIRI	614		12.6 (11.07–13.11)	4.67 (4.21–5.36)	11.1	
Tabernero, J. et al. (2015) [7]	RAM + FOLFIRI	536	OS	13.3 (12.4–14.5)	5.7 (5.5–6.2)	13.4	OS: (0.844; 95%CI 0.73–0.976; *p* = 0.0219)
	FOLFIRI	536		11.7 (10.8–12.7)	4.5 (4.2–5.4)	12.5	

**Table 4:** Toxicity and cost of second-line treatments with angiogenesis inhibitors.

	BEV + FOLOX/FOLFIRI			RAM + FOLFIRI			AFL + FOLFIRI		
	(ML18147)			(RAISE)			(VELOUR)		
Adverse Event (%)	Grade ≥ 3	Grade 4	Grade 5	Grade ≥ 3	Grade 4	Grade 5	Grade ≥ 3	Grade 4	Grade 5
Hypertension	2	-	0	11.2	0.2	0	19.3	0.2	-
Proteinuria	<1	-	0	3	0.2	0	7.8	0.3	-
Hemorrhage	2	-	0.2	5.5	0.4	1,2	3.0	0.2	-
GI perforation	2	-	0.2	1.7	1.0	1.0	0.5	0.3	-
Arterial thromboembolic event	1	-	0.2	0.8	0.2	0.2	1.8	1.0	-
Venous thromboembolic event	5	-	0	4.2	1.0	0	7.8	4.7	-
Healing complications	<1	-	0	0.2	-	-	-	-	-
Treatment-related death (%)	0.98			1.49			1		
Cost in Japan, for 1 course									
Height 160 cm, weight 50 kg, body surface area 1.46 m^2^	BEV+FOLFIRI			RAM+FOLFIRI			AFL+FOLFIRI		
	¥143,206			¥338,621			¥226,712		

**Table 5:** Summary of biomarkers of angiogenesis inhibitors.

Clinical Trial	Study Design	Treatment Line	Biomarker	Sample Used for Biomarker Assessment	Biomarker Level	Treatment Arm	*n*	Median OS, Months (95%CI)	HR (95%CI)	Interaction *p*	Median PFS, Months (95%CI)	HR (95%CI)	Interaction *p*
Bai L et al. (2015) [28]	Retrospective	1st line	VEGF-A	Plasma	all	BEV + CT	91	26.6 (20.6–32.5)			10.5 (9.1–11.8)		
						CT	95	24.5 (20.5–28.4)			6.9 (5.7–8.1)		
					Low	BEV + CT	62	50	0.42 (0.2-.0.84) *p* = 0.011	0.034	10.8	0.53 (0.36–0.76) *p* = 0.01	0.023
						CT	61	28			6.4		
					High	BEV + CT	33	19.6	1.25 (0.77–2.02) *p* = 0.370		7.8	0.90 (0.53–1.53) *p* = 0.70	
						CT	27	29.9			7.8		
Parikh, A.R. et al. (2019) [29]	Phase II	1st line	VEGF-A	Plasma	Low	BEV + CT	185	27.9 (24.97–36.01)	1.64 (1.20–2.24) *p* < 0.01				
					High		184	22.8 (18.76–27.27)					
Okamoto, W. et al. (2022) [30]	Phase II	1st line	VEGF-Asi	Plasma	Low	BEV + CT	48	38	1.7 (1.1–2.9) *p* = 0.029		13	1.3 (0.8–2.1) *p* = 0.25	
					High		49	26			11		
Weickhardt, A.J. et al. (2015) [31]	Post hoc analysis of MAX trial (Phase III)	1st line	VEGF-D	Tumor tissue (Immunohistochemical analysis.)	score 0.1+	BEV + CT	32	not reached	0.35 (0.13–0.90)	0.01	16.8	0.22 (0.08–0.55)	0.02
						CT		18.9			5.8		
					score 2	BEV + CT	117	21.6	0.82 (0.52–1.30)		8.8	0.67 (0.45–1.0)	
						CT		20.6			6		
					score 3	BEV + CT	110	19.4	1.28 (0.79–2.09)		9	0.77 (0.50–1.17)	
						CT		24.5			7		
Tabernero, J. et al. (2018) [32]	Planned study of RAISE (Phase III)	2nd line	VEGF-D	Plasma	Low	RAM + FOLFIRI	176	12.6 (10.7–14.0)	1.32 (1.02–1.70) *p* = 0.0344	0.0005	5.4 (4.2–5.8)	1.16 (0.93–1.45) *p* = 0.1930	<0.0001
						FOLFIRI	172	13.1 (11.8–17.0)			5.6 (5.3–6.9)		
					High	RAM + FOLFIRI	270	13.9 (12.5–15.6)	0.73 (0.60–0.89) *p* = 0.0022		6.0 (5.6–7.0)	0.62 (0.52–0.74) *p* < 0.0001	
						FOLFIRI	266	11.5 (10.1–12.4)			4.2 (4.1–4.5)		
Van Cutsem, E. et al. (2020) [34]	Post hoc analysis of VELOUR trial (Phase III)	2nd line	VEGF-A	Plasma	Low	AFL + FOLFIRI	148	12.8 (11.6–16.7)	0.974 (0.735–1.291) *p* = 0.854		6.8 (5.9–7.5)	0.898 (0.681–1.185) *p* = 0.448	
						FOLFIRI	139	12.9 (11.1–15.1)			5.5 (4.5–6.7)		
					High	AFL + FOLFIRI	140	12.5 (10.4–15.6)	0.673 (0.508–0.892) *p* = 0.00566		6.9 (5.7–8.3)	0.660 (90.493–0.885) *p* = 0.00521	
						FOLFIRI	126	9.6 (8.5–11.3)			4.0 (3.0–4.3)		

**Table 6:** Comparative study of anti-EGFR antibodies and BEV after BEV failure.

Anti-EGFR Antibody versus Bevacizumab beyond Bevacizumab										
Clinical Trial	Phase	Previous Regimen	RAS Status	Treatment Arm	*n*	Primary Endpoint	Median OS, Months (95%CI)	Median PFS, Months (95%CI)	ORR% (95%CI)	HR
Hecht, J.R. et al. (2015) [67]	II	Oxaliplatin-based chemotherapy with BEV	*KRAS* wild type	Panitumumab + FOLFIRI	91	PFS	18 (13.5–21.7)	7.7 (5.7–11.8)	32 (23–43)	PFS: (1.01; 95%CI: 0.68–1.50; *p* = 0.97)
				Bevacizumab + FOLFIRI	91		21.4 (16.5–24.6)	9.2 (7.8–10.6)	19 (11–29)	
Shitara, K. et al. (2016) [68]	II	Fluoropyrimidine + oxaliplatin + BEV	*KRAS* exon 2 (codon 12 or 13) wild type	Panitumumab + FOLFIRI	59	OS	16.2 (12.5–22.4)	6.0 (5.0–7.5)	46.2 (32.2–60.5)	OS: (1.16; 95%CI: 0.76–1.77)
				Bevacizumab + FOLFIRI	58		13.4 (11.1–1.77)	5.9 (3.9–7.8)	5.7 (1.2–15.7)	
Bennouna, J. et al. (2019) [69]	II	BEV + FOLFOX or FOLFIRI	*KRAS*(exons 2, 3, 4) *NRAS*(exons 2, 3, 4) wild type	Cetuximab + FOLOX or FOLFIRI	65	PFS rate at 4 months	10.4 (7.0–16.2)	5.6 (4.2–6.5) 66.7% (53.6–76.8)	31.8 (20.3–43.2)	PFS: (0.71; 95%CI: 0.50–1.02; *p* = 0.06)
				Bevacizumab + FOLOX or FOLFIRI	67		15.8 (9.5–22.3)	7.1 (5.7–8.2) 80.3% (68.0–88.3)	24.6 (14.1–35.1)	
